# Dosimetric study of uniform scanning proton therapy planning for prostate cancer patients with a metal hip prosthesis, and comparison with volumetric‐modulated arc therapy

**DOI:** 10.1120/jacmp.v15i3.4611

**Published:** 2014-05-08

**Authors:** Suresh Rana, ChihYao Cheng, Yuanshui Zheng, Wen Hsi, Omar Zeidan, Niek Schreuder, Carlos Vargas, Gary Larson

**Affiliations:** ^1^ Department of Medical Physics ProCure Proton Therapy Center Oklahoma City OK; ^2^ Department of Radiation Oncology Vantage Oncology West Hills CA; ^3^ Department of Medical Physics McLaren Cancer Institute Flint MI; ^4^ Department of Radiation Physics UF Health Cancer Center – Orlando Health Orlando FL; ^5^ Department of Medical Physics Provision Center for Proton Therapy Knoxville TN; ^6^ Radiation Oncology Proton Collaborative Group (PCG) Bloomington IN; ^7^ Department of Radiation Oncology ProCure Proton Therapy Center Oklahoma City OK USA

**Keywords:** uniform scanning proton therapy, VMAT, metallic hip prosthesis, prostate cancer

## Abstract

The main purposes of this study were to 1) investigate the dosimetric quality of uniform scanning proton therapy planning (USPT) for prostate cancer patients with a metal hip prosthesis, and 2) compare the dosimetric results of USPT with that of volumetric‐modulated arc therapy (VMAT). Proton plans for prostate cancer (four cases) were generated in XiO treatment planning system (TPS). The beam arrangement in each proton plan consisted of three fields (two oblique fields and one lateral or slightly angled field), and the proton beams passing through a metal hip prosthesis was avoided. Dose calculations in proton plans were performed using the pencil beam algorithm. From each proton plan, planning target volume (PTV) coverage value (i.e., relative volume of the PTV receiving the prescription dose of 79.2 CGE) was recorded. The VMAT prostate planning was done using two arcs in the Eclipse TPS utilizing 6 MV X‐rays, and beam entrance through metallic hip prosthesis was avoided. Dose computation in the VMAT plans was done using anisotropic analytical algorithm, and calculated VMAT plans were then normalized such that the PTV coverage in the VMAT plan was the same as in the proton plan of the corresponding case. The dose‐volume histograms of calculated treatment plans were used to evaluate the dosimetric quality of USPT and VMAT. In comparison to the proton plans, on average, the maximum and mean doses to the PTV were higher in the VMAT plans by 1.4% and 0.5%, respectively, whereas the minimum PTV dose was lower in the VMAT plans by 3.4%. The proton plans had lower (or better) average homogeneity index (HI) of 0.03 compared to the one for VMAT (HI = 0.04). The relative rectal volume exposed to radiation was lower in the proton plan, with an average absolute difference ranging from 0.1% to 32.6%. In contrast, using proton planning, the relative bladder volume exposed to radiation was higher at high‐dose region with an average absolute difference ranging from 0.4% to 0.8%, and lower at low‐ and medium‐dose regions with an average absolute difference ranging from 2.7% to 10.1%. The average mean dose to the rectum and bladder was lower in the proton plans by 45.1% and 22.0%, respectively, whereas the mean dose to femoral head was lower in VMAT plans by an average difference of 79.6%. In comparison to the VMAT, the proton planning produced lower equivalent uniform dose (EUD) for the rectum (43.7 CGE vs. 51.4 Gy) and higher EUD for the femoral head (16.7 CGE vs. 9.5 Gy), whereas both the VMAT and proton planning produced comparable EUDs for the prostate tumor (76.2 CGE vs. 76.8 Gy) and bladder (50.3 CGE vs. 51.1 Gy). The results presented in this study show that the combination of lateral and oblique fields in USPT planning could potentially provide dosimetric advantage over the VMAT for prostate cancer involving a metallic hip prosthesis.

PACS number: 87.55.D‐, 87.55.ne, 87.55.dk

## I. INTRODUCTION

Recently, there has been growing interest in treating prostate cancer using protons, which have finite range in tissue, sharp lateral penumbra, and near‐zero exit dose when compared to conventional MV X‐rays (photons).^(1)^ Since the majority of the proton dose can be deposited in a region called the spread‐out Bragg peak (SOBP) with sharp dose falloff after SOBP, proton therapy can spare the critical structures that are adjacent to the target volume. Prostate cancer is the most commonly diagnosed cancer among American men.^(2)^ Prostate cancer patients with a metallic hip prosthesis are rare at our proton therapy center. Metallic hips are composed of high‐Z materials, which produce streak artifacts on the computed tomography (CT) images, and this provides a challenge in contouring the organs accurately. Furthermore, due to the limitation of commercially available treatment planning systems (TPSs) in beam modeling, significant errors in dose calculations may occur near and beyond the metallic hips.^3^, ^4^, ^5^ Thus, beam entrance through metallic devices is not recommended in external‐beam radiation therapy (EBRT) planning.^(5)^

Several researchers have investigated proton therapy planning of prostate cancer using mostly parallel opposed lateral fields or slightly angled fields.^6^, ^7^, ^8^, ^9^, ^10^, ^11^, ^12^, ^13^, ^14^, ^15^, ^16^ For prostate cases that do not involve a metallic hip prosthesis, we generally use two parallel opposed lateral fields for proton planning at ProCure Proton Therapy Center, Oklahoma City. However, if a prostate cancer patient has a metallic hip prosthesis, the proton treatment plan can no longer have the second lateral field because proton beam passing through metallic hip prosthesis is not recommended.^(5)^ Thus, a proton prostate plan involving a metallic hip prosthesis requires the beam setup to include an angled beam too, but such beam arrangement may lead to an increase in dose to the critical structures depending on the orientation of the angled beam. Trofimov et al.^(16)^ have shown that slightly anteriorly angled lateral proton beam can reduce the volume of the rectum in the high‐dose region. Recently, Tang et al.^(17)^ demonstrated that anterior‐oblique beams can significantly reduce dose to the anterior rectal wall, particularly at high‐dose levels, when compared to beam arrangement having two parallel opposed lateral proton fields.

Although a number of studies have investigated the treatment planning techniques of prostate cases that involve metallic hips in external‐beam photon radiation therapy,^18^, ^19^, ^20^, ^21^ proton therapy planning for prostate cancer patients with a metallic hip prosthesis, especially for uniform scanning proton therapy (USPT), remains to be addressed. At the ProCure Proton Therapy Center, we use USPT to treat all cancer patients, and other beam delivery systems, such as double scatter and pencil beam scanning, are not currently implemented in our proton therapy unit. Since the current literature on proton therapy lacks the dosimetric data for prostate cases involving prosthesis, we aim to investigate the dosimetric quality of USPT planning using three fields for prostate cancer patients with a metallic hip prosthesis. Comparative studies on prostate cancer in external‐beam photon radiation therapy^22^, ^23^ have shown that VMAT could achieve dose distributions comparable to that of intensity‐modulated radiation therapy (IMRT), and the VMAT requires shorter delivery time compared to the IMRT. For these reasons, there is a growing interest in using the VMAT for the prostate cancer compared to the IMRT. Since USPT and VMAT are considered two relatively new modalities to treat the prostate cancer, we have also compared the dosimetric results between the proton plans and VMAT plans.

## II. MATERIALS AND METHODS

In this retrospective study, four prostate cases involving a metallic hip prosthesis were included. All four patients have consented to participation in the Proton Collaborative Group research study (REG01‐09, WIRB Protocol #20091082). The treatment plans of all four prostate cases were clinically approved and delivered using USPT at ProCure Proton Therapy Center, between February 2012 and March 2013.

### A. Simulation and contouring

**Figure 1:**
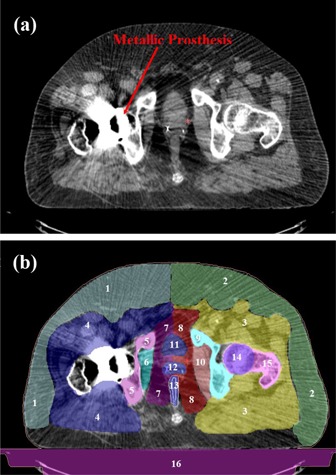
A transversal view of the computed tomography (CT) slice (case #4) showing (a) streaking artifacts produced by a metallic hip prosthesis, and (b) contoured artifacts and structures, which are numbered from 1 to 16. Details of each structure (1‐16) and their relative stopping power values are provided in Table 1.

Prior to CT simulation, all four patients underwent VisiCoil linear fiducial markers (IBA Dosimetry, Schwarzenbruck, Germany) placement within the prostate. The CT simulation was performed in a supine position with a full bladder while patients were immobilized using vac‐lok system (CIVCO Medical Solutions, Kalona, IA). The CT images were acquired with 1.25 mm spacing using General Electric CT Scanner (GE Healthcare, Waukesha, WI). The CT dataset was imported into VelocityAI, version 2.8.0 (Velocity Medical Solutions, Atlanta, GA) for contouring purpose. The planning CT was then fused with the patient's magnetic resonance imaging (MRI) scan, which helped in visualizing and contouring the organs on the CT slices. The clinical target volume (CTV) was defined as the prostate and seminal vesicles, whereas the planning target volume (PTV) was determined by expanding the CTV (3 mm to the posterior and 4 mm elsewhere to the CTV). The delineation of organs at risk (OARs) (rectum, bladder, and right or left femoral head), and other relevant structures was done. Since image artifacts in the planning CT can affect the dose calculations, all streaking artifacts were contoured prior to the treatment planning phase (Fig. 1). In the case of proton plans, the artifacts were overridden by appropriate relative stopping power values (Table 1), which were obtained by sampling the tissues in the same CT dataset. The air in the rectum was also overridden with relative stopping power of 1.0. Similarly, in the case of VMAT plans, all contoured artifacts representing soft tissue were overridden with unit density. Density/relative stopping power override was not done for the metallic prosthesis, since beam entry through it was avoided in the planning.

**Table 1:** Contoured artifacts and structures (Fig. 1), with their relative stopping power values, for dose calculations in proton plans

*Number*	*Structure*	*Relative Stopping Power*
1	Left Abdominal Fat	0.95
2	Right Abdominal Fat	0.94
3	Right Abdominal Muscle	1.05
4	Left Abdominal Muscle	1.05
5	Left Pelvic Bone	1.05
6	Left Pelvic Muscle	1.05
7	Left Pelvic Fat	0.95
8	Right Pelvic Fat	0.95
9	Right Pelvic Bone	1.05
10	Right Pelvic Muscle	1.05
11	Bladder	1.00
12	Prostate	1.04
13	Rectum	1.00
14	Right Femoral Head	1.10
15	Right Femoral Bone	1.10
16	Table	0.15

### B. Proton planning

Proton plans were generated in the XiO TPS (CMS Inc., St. Louis, MO) for the treatment to be delivered in the inclined or gantry room. The XiO TPS uses the uniform scanning proton beam commissioning data that were measured at our proton therapy center. In USPT, the degraded proton beam is scanned laterally with a constant frequency in order to deliver a uniform dose for a near rectangular scanning area. A detailed description on the USPT system has been provided elsewhere.^(24)^

**Figure 2:**
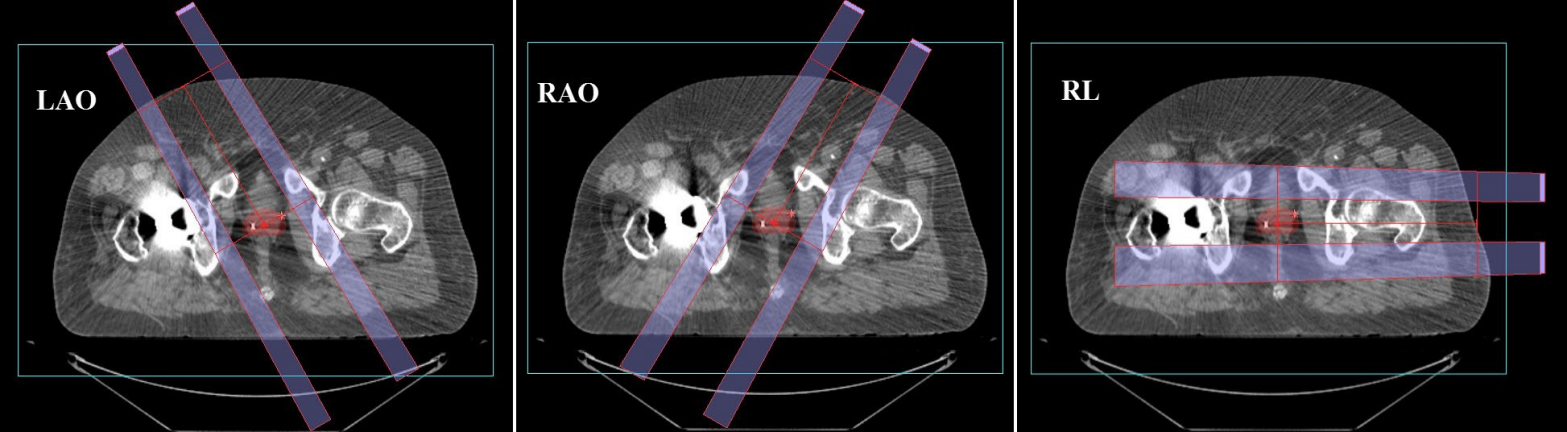
A transversal view of proton beams arrangement for the prostate treatment plan (case #4) in the XiO treatment planning system. The figure shows three fields: left anterior–oblique (LAO) with gantry angle 30° and couch angle 180° (left panel), right anterior–oblique (RAO) with gantry angle 30° and couch angle 0° (middle panel), and right lateral (RL) with gantry angle 90° and couch angle 0° (right panel).

The proton treatment plans were set up using parameters provided in Table 2. For example, Fig. 2 shows the beam arrangement for case #4, which had three fields: left anterior oblique (LAO) with gantry angle 30° and couch angle 180°, right anterior oblique (RAO) with gantry angle 30° and couch angle 0°, and right lateral (RL) with gantry angle 90° and couch angle 0°. In all four cases, proton beams passing through the metal hip prosthesis was avoided, and isocenter of all three beams in each plan was placed at the center of the PTV. The aperture margin was selected based on the lookup table using penumbra for the proton range of each beam. A total of 79.2 cobalt gray equivalent (CGE) dose was prescribed to the PTV with daily fraction of 1.8 CGE. The doses in proton treatment plans were calculated using relative biologic effectiveness (RBE) of 1.1.

**Table 2:** Parameters used for the beam set up in the proton plans

*Case #*	*Field Orientation*	*Gantry/Couch Angle*	*Beam Weighting*	*Delivery Schema*	*PTV Coverage* ^a^
	LPO	$265^{\circ }/0^{\circ }$	50%	Daily	
1	RAO	$10^{\circ }/0^{\circ }$	25%	Every Other Day	94.88%
	RPO	$145^{\circ }/0^{\circ }$	25%	Every Other Day	
	LL	$270^{\circ }/0^{\circ }$	50%	Daily	
2	RAO	$30^{\circ }/0^{\circ }$	25%	Every Other Day	94.21%
	RPO	$150^{\circ }/0^{\circ }$	25%	Every Other Day	
	LL	$90^{\circ }/180^{\circ }$	50%	Daily	
3	LAO	$30^{\circ }/180^{\circ }$	25%	Every Other Day	94.73%
	RAO	$30^{\circ }/0^{\circ }$	25%	Every Other Day	
	LAO	$30^{\circ }/180^{\circ }$	33.3%	Every Other Day	
4	RAO	$30^{\circ }/0^{\circ }$	33.3%	Every Other Day	98.97%
	RL	$90^{\circ }/0^{\circ }$	33.3%	Daily	

a PTV coverage (relative volume of the PTV receiving the prescription dose) value from the proton plan was used for the dose‐volume normalization in the VMAT plan for the corresponding case.

LPO=left posterior−oblique; RAO=right anterior−oblique; RPO=right posterior‐oblique; LL=left lateral; LAO=left anterior−oblique; RL=right lateral.

The proton beam delivery schema (Table 2) was selected such that the lateral beam (cases #2, #3, and #4) or slightly angled beam (case #1) was delivered daily, whereas two oblique beams in each case were delivered every other day. The weighting of a slightly angled beam (case #1) or the lateral beam (cases #2 and #3) passing through the femoral head was selected twice that of an oblique beam (Table 2). The beam with more weighting is typically associated with a higher number of monitor units. Due to range uncertainty in USPT, it is recommended to avoid the heavily weighted beam directing towards the OAR, especially when target volume is abutting the OAR. Since rectum and bladder are two most important critical structures in prostate cancer treatment, the direction of the 50% weighted beam was selected such that its distal end does not fall inside the OAR. However, if the treatment plan with the 50% weighting on the lateral beam results in violation of dose constraints for the femoral head, all three beams (lateral and two oblique) were equally weighted, such as for case #4 in this study (Table 2).

Dose calculations were performed using the pencil beam algorithm^(25)^ with dose calculation grid size of $3\times 3\times 3\;{\text{mm}}^{3}$. The aperture margin, proton range, and modulation of each beam were manually adjusted with an aim of minimizing dose to the OARs and meeting our planning objectives: 1) minimum dose to the PTV was ≥75.2 CGE (i.e., 95% of the prescription dose); 2) at least 95% of the PTV volume received 79.2 CGE; 3) 98% isodose line was away from the PTV by ≥5mm at the distal end; 4) 98% isodose line was away from the PTV by ≥ 5 mm at the proximal end; and 5) 60% isodose line was away from the femoral head. Furthermore, the range compensators of each beam were generated with smearing radius of 1.2 cm to account patient setup and organ motion uncertainties. From the final proton treatment plan of each case, the relative volume of the PTV receiving total prescription dose was recorded, and this PTV coverage value was used for dose‐volume normalization in the VMAT plan of the corresponding prostate case.

### C. VMAT planning

The Digital Imaging and Communications in Medicine (DICOM) CT images that were used for proton planning were deidentified before transferring them to West Hills Radiation Therapy Center, Vantage Oncology, CA, for VMAT planning. The DICOM CT dataset and contours were imported in the Eclipse TPS, version 11.01 (Varian Medical Systems, Palo Alto, CA) to ensure the integrity during data transfer. The VMAT is also referred as RapidArc in the Eclipse TPS. The VMAT system can deliver a highly conformal radiation dose to the target using one or more arcs, and the delivery technique allows the simultaneous variation of gantry rotation speed, dose rate, and multileaf collimator (MLC) leaf positions.^(26)^

**Figure 3:**
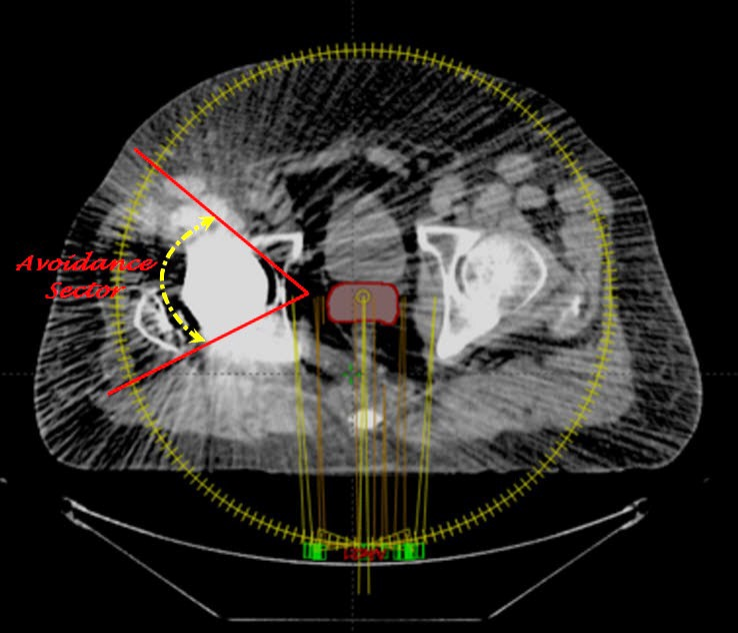
A transversal view of the VMAT plan setup for prostate treatment plan in Eclipse treatment planning system showing an avoidance sector (case #4).

The VMAT planning was done using Varian Clinac iX 6 MV X‐ray beams, and VMAT plans of all four cases were created for total dose of 79.2 Gy prescribed to the PTV with a daily dose of 1.8 Gy. Figure 2 shows an example of VMAT plan setup using Varian Standard Scale in the Eclipse TPS, with the first arc in a clockwise direction (arc angle: $359^{\circ }\to 1^{\circ }$; collimator angle: 135°) and the second arc in a counterclockwise direction (arc angle: $1^{\circ }\to 159^{\circ }$; collimator angle: 225°). The beam's‐eye‐view graphics in the Eclipse TPS were used to determine the avoidance sector (Fig. 3) that would avoid the radiation beam entrance through the metallic hip prosthesis; however, the beams were allowed to deliver exit dose to the prosthesis.

The isocenter of all the VMAT plans was placed at the center of the PTV. The VMAT plans were then optimized using progressive resolution optimizer, version 11.1, in the Eclipse TPS. During the optimization process, dose‐volume constraints and weightings of the PTV and OARs were adjusted to achieve the dose‐volume objectives of proton planning. Hot spot was not allowed to occur within the PTV overlapping the OARs. The optimized VMAT plans were computed with anisotropic analytical algorithm (AAA), version 11.1, using 2.5 mm dose calculation grid size. The calculated VMAT plan of each case was then normalized using the PTV coverage value that was obtained from the proton planning (Table 2).

### D. Plan evaluation

#### D.1 Dose‐volume histogram analysis

The dose‐volume histograms (DVHs) of calculated treatment plans were used to evaluate the dosimetric quality of the USPT and VMAT. For the PTV, the analysis was done for the minimum dose, maximum dose, mean dose, homogeneity index (HI) (defined in Eq. (1)), and relative volume irradiated to 95%, 103%, 105%, and 107% ($V_{95\%},V_{103\%},V_{105\%}$, and $V_{107\%}$, respectively). For the rectum and bladder, the mean dose and the relative volumes that received 80, 70, 60, 50, 40, 30, 20, and 10 CGE or Gy ($V_{80},V_{70},V_{60},V_{50},V_{40},V_{30},V_{20}$, and $V_{10}$, respectively) were compared. Additionally, dose to the femoral head was obtained for comparison.

(1) $$HI=\frac{\left(D_{5\%}-D_{95\%}\right)}{\text{Prescription Dose}}$$

where $D_{5\%}$ and $D_{95\%}$ represent doses to 5% and 95% of the PTV, respectively. A smaller HI value means better dose homogeneity within the PTV volume.

#### D.2 Equivalent uniform dose analysis

Equivalent uniform dose (EUD) evaluation was performed using the DVHs of the treatment plans, which were exported from the Eclipse and XiO TPSs using the dose bin size of 50 cGy. Specifically, we utilized the MATLAB program^(27)^ (The MathWorks, Natick, MA) and the DVHs of the treatment plans (VMAT and protons) to calculate the EUD, which is based on the Niemierko's phenomenological model.^27^, ^28^

The EUD^27^, ^28^ is defined as:

(2) $$EUD={\left(\sum_{i=1}\left(v_{i}EQD_{i}^{a}\right)\right)}^{\frac{1}{a}}$$

(3) $$EQD=D\times \frac{\left(\frac{\alpha }{\beta }+\frac{D}{n_{f}}\right)}{\left(\frac{\alpha }{\beta }+2\right)}$$

In Eq. (2), a is a unit‐less model parameter that is specific to the normal structure or tumor of interest, and $v_{i}$ is unit less and represents the ith partial volume receiving dose $D_{i}$ in Gy.^27^, ^28^ Since the relative volume of the whole structure of interest corresponds to 1, the sum of all partial volumes $v_{i}$ will equal 1.^27^, ^28^ The EQD is the biologically equivalent physical dose of 2 Gy. In Eq. (3), $n_{f}$ and $d_{f}=D/n_{f}$ are the number of fractions and dose per fraction size of the treatment course, respectively. The α/β is the tissue‐specific linear quadratic (LQ) parameter of the organ being exposed. The EUD calculations in this study were based on the parameters listed in Table 3.^29^, ^30^

**Table 3:** Parameters used to calculate the equivalent uniform dose (EUD) using Niemierko's method

*Tissue*	*Volume Type*	*100°% dpf*	*#f*	*a*	*Dpf(Gy)*	α/β *(Gy)*
Prostate	Tumor	1.8	44	−10	2	1.5
Rectum	Normal	1.8	44	5	2	8
Bladder	Normal	1.8	44	7	2	3
Femoral head	Normal	1.8	44	4	2	0.85

100%dpf=100% dose per fraction; #f=number of fractions; α=unit‐less model parameter that is specific to the normal structure or tumor of interest; α/β=alpha−beta ratio; dpf=parameters' source data's dose per fraction (the parameters are obtained from Zeng et al.^(29)^ and Kehwar^(30)^).

## III. RESULTS

### A. PTV

Table 4 shows the dosimetric results for the PTV. Both the proton plans and VMAT plans showed that the relative PTV volume receiving at least 95% of the prescription dose was 100%. In comparison to the proton plans, on average, the maximum and mean doses to the PTV were slightly higher in the VMAT plans by 1.4% and 0.5%, respectively, whereas the minimum PTV dose was lower in the VMAT plans by 3.4%. These results for the PTV doses demonstrated that the proton plans produced values closer to the prescription dose. Furthermore, a smaller

**Table 4:** Comparison of the dosimetric parameters of PTV for the VMAT and proton plans. The values are averaged over four analyzed cases. (Note: PTV coverage was identical in the VMAT and proton plans for a given prostate case.)

*PTV (volume: 86.4 cc)*	*VMAT*	*Proton*
Minimum Dose	73.4 Gy	76.0 CGE
Maximum Dose	84.0 Gy	82.8 CGE
Mean Dose	81.1 Gy	80.7 CGE
$D_{95\%}$	79.4 Gy	79.3 CGE
$V_{95\%}(\%)$	100	100
$V_{103\%}(\%)$	26.6	14.1
$V_{105\%}(\%)$	1.6	0
$V_{107\%}(\%)$	0	0
HI	0.04	0.03

PVT=planning target volume; $V_{x\%}=$ relative volume of the PTV receiving x% of the prescription dose; $D_{95\%}=\text{dose at}\;95\%\;\text{of the PTV}$; HI=homogeneity index.

HI value in the proton plans (HI=0.03) indicates that the proton plans produced better dose homogeneity within the PTV compared to the VMAT plan (HI=0.04). The $V_{103\%}$ and $V_{105\%}$ were lower in the proton plans.

### B. Rectum

**Figure 4:**
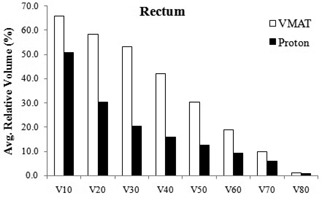
Relative volume of rectum receiving 10–80 CGE or Gy $\left(V_{10-80}\right)$ in the VMAT and proton plans The values are averaged over four analyzed cases. (Note: PTV coverage was identical in the VMAT and proton plans for a given prostate case.)

Table 5 shows the dosimetric results for the rectum. The mean dose to the rectum was lower in the proton plans by average difference of 45.1%, compared to the one in the VMAT plans. Similarly, the rectal volume receiving 10–80 CGE was always lower in the proton plans by an average absolute difference of 16.6%±11.8% (range, 0.1%–32.6%) (Fig. 4). Both the VMAT and proton planning produced higher volumes of rectum being irradiated to low doses compared to high doses.

**Table 5:** Comparison of the dosimetric parameters of rectum for the VMAT and proton plans. The values are averaged over four analyzed cases. (Note: PTV coverage was identical in the VMAT and proton plans for a given prostate case.)

*Rectum (volume: 118.3 cc)*	*VMAT*	*Proton*
Mean Dose	32.9 Gy	18.1 CGE
$V_{30}(\%)$	53.1	20.4
$V_{50}(\%)$	30.3	12.5
$V_{70}(\%)$	9.8	6.0

$V_{x}=$ relative volume of the rectum receiving × Gy or CGE.

### C. Bladder

**Figure 5:**
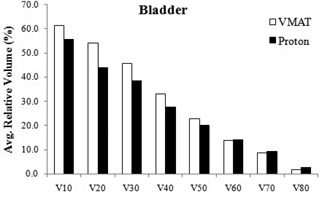
Relative volume of bladder receiving 10‐80 CGE or Gy $\left(V_{10-80}\right)$ in the VMAT and proton plans The values are averaged over four analyzed cases. (Note: PTV coverage was identical in the VMAT and proton plans for a given prostate case.)

Table 6 shows the dosimetric results for the bladder. The mean dose to the bladder was lower in the VMAT plans compared to that in the proton plans, with an average difference of 22.0%. However, in contrast to the results for the rectum, the proton planning produced higher $V_{60}-V_{80}$ and lower $V_{10}-V_{50}$ for the bladder (Fig. 5). Specifically, the average absolute differences in $V_{60}-V_{80}$ and $V_{10}-V_{50}$ were 6.1%±2.7% and 0.6%±0.2%, respectively. Both the VMAT and proton planning resulted in higher volumes of bladder being irradiated to low doses compared to high doses.

**Table 6:** Comparison of the dosimetric parameters of bladder for the VMAT and proton plans. The values are averaged over four analyzed cases. (Note: PTV coverage was identical in the VMAT and proton plans for a given prostate case.)

*Bladder (volume: 257.3 cc)*	*VMAT*	*Proton*
Mean Dose	28.4 Gy	23.9 CGE
$V_{30}(\%)$	45.5	38.5
$V_{50}(\%)$	22.7	20.0
$V_{70}(\%)$	8.6	9.4

a $V_{x}=$ relative volume of the rectum receiving × Gy or CGE.

### D. Femoral head

Table 7 shows the dosimetric results for the femoral head. In comparison to the VMAT plan, the mean dose to the femoral head was higher in the proton plans by an average difference of 79.6%. Similarly, the results in the proton plans were higher both at $V_{15}$ with an average absolute difference of 19.9%, and at $V_{20}$ with an average absolute difference of 26.1%.

**Table 7:** Comparison of the dosimetric parameters of femoral head for the VMAT and proton plans. The values are averaged over four analyzed cases. (Note: PTV coverage was identical in the VMAT and proton plans for a given prostate case.)

*Femoral Head (volume: 80.38 cc)*	*VMAT*	*Proton*
Mean Dose	16.8 Gy	23.9 CGE
$V_{15}(\%)$	55.9	75.9
$V_{20}(\%)$	33.7	59.9

$V_{x}$ relative volume of the rectum receiving × Gy or CGE.

### E. EUD

Table 8 shows the EUD results for the prostate tumor, rectum, bladder, and femoral head. In comparison to the VMAT, the proton planning produced lower EUDs for the rectum (43.7 CGE vs. 51.4 Gy) and higher EUD for the femoral head (16.7 CGE vs. 9.5 Gy), whereas both the VMAT and proton planning produced comparable EUDs for the prostate tumor (76.2 CGE vs. 76.8 Gy) and bladder (50.3 CGE vs. 51.1 Gy).

**Table 8:** Comparison of the equivalent uniform dose (EUD) in the VMAT and proton plans. The values are averaged over four analyzed cases. (Note: PTV coverage was identical in the VMAT and proton plans for a given prostate case.)

	*EUD*	
*Structure*	*VMAT*	*Proton*
Prostate	76.8 Gy	76.2 CGE
Rectum	51.4 Gy	43.7 CGE
Bladder	51.1 Gy	50.3 CGE
Femoral Head	9.5 Gy	16.7 CGE

## IV. DISCUSSION

In this study, we compared the dosimetric quality of the USPT and VMAT for prostate cancer patients with a metallic hip prosthesis, and the dosimetric evaluation was done by applying identical PTV coverage in the proton and VMAT plans of the corresponding case. For a prostate cancer case without metallic hip, we generally use two parallel opposed lateral proton beams for treatment planning at our proton therapy center. In this study, we demonstrated the dosimetric quality of USPT using three fields to treat the prostate cancer patients with a single metallic hip prosthesis. During the proton treatment planning phase of all four cases, different arrangement of oblique proton fields were investigated, and we have used the optimal treatment technique that can be implemented in the treatment rooms of our proton therapy center.

The PTV doses examined in this study showed that the proton planning produced values closer to the prescription dose with slightly better dose homogeneity when compared to the VMAT planning. The lower maximum PTV dose in the proton plans suggests that smaller hot spots can be achieved in prostate plans using proton beams, and this could translate into lower urethral doses and minimize the risk of urethral symptoms and bladder neck.^(13)^

The major dosimetric advantage of using uniform scanning proton beams was demonstrated for the rectum as its dosimetric results in the proton plans were significantly lower in the low‐, intermediate‐, and high‐dose regions, when compared to the ones in the VMAT plans. The rectal EUD was also lower in the protons plans. Such dosimetric benefit for rectum was likely due to customized aperture margins minimizing dose to the rectum, compensator design conforming the dose distally, sharp lateral penumbra, and sharp dose falloff after SOBP for the anterior–oblique fields. The proton plans showed lower exposure of bladder in the low‐ and intermediate‐dose regions, but higher exposure of bladder in the high‐dose region when compared to the results in the VMAT plans. However, results for bladder in the high‐dose region of protons plans are within the dose constraints ($V_{65},V_{70}$, and $V_{75}$ should be less than 50%, 35%, and 25%, respectively) recommended by QUANTEC study.^(31)^

The results presented in this study show that proton therapy can be used to treat the complex prostate cases involving a metallic hip prosthesis. Proton therapy, however, has several uncertainties such as uncertainty in the proton beam range. A review article by Paganetti^(32)^ showed that there is no common consensus on the use of range uncertainty among all proton centers in the US. For instance, the University of Florida Proton Therapy Institute uses the range uncertainty 2.5%+1.5mm, the Massachusetts General Hospital uses the range uncertainty 3.5%+1mm, and the Roberts Proton Therapy Center at the University of Pennsylvania, MD Anderson Proton Therapy Center, and Loma Linda University Medical Center use range uncertainty 3.5%+3mm.^(32)^ The range uncertainty guideline at our proton center is 2.5%+2.0mm. Proton treatment planning margin incorporating a larger range uncertainty value may overdose the OAR abutting the target volume. However, it is also critical not to underdose the tumor by underestimating the range uncertainty in proton treatment planning. Although recommended range uncertainty at our center is 2.5%+2mm, this value may require an adjustment depending on the beam orientation, patient geometry, and location of the OARs with respect to the tumor volume. On average, the range uncertainty used in this study was about 2.5%+3mm, which is 1 mm more than the recommended value at our center. Beam‐specific PTV margins based on the range uncertainly of each beam were not used in this study. In the near future, we aim to investigate the dependency of range uncertainty on the treatment planning system, tumor site, and treatment delivery unit.

One of the drawbacks of VMAT planning involving a metallic hip prosthesis is the reduced number of available beam angles, since primary beams passing through the metallic hip are avoided. Such limitation causes the dose streaking in the anterior‐posterior and posterioranterior directions, thus increasing dose to the bladder and rectum. The DVH results presented in this study showed that the VMAT technique produced less favorable dosimetric results, except for the femoral head and the bladder in high‐dose region. The tight dose constraints placed on the OARs can force the TPS to lower the dose to the OARs but for the reduced PTV coverage and decreased dose homogeneity across the PTV. Thus, use of different dose‐volume constraints for the PTV and OARs during the VMAT plan optimization may yield dosimetric results that are different from the ones presented in this study.

Literature on proton treatment planning of prostate cancer cases involving a metallic hip prosthesis is currently not available. Nevertheless, it is relevant to mention treatment planning studies done by other researchers using proton beams. For example, Chera et al.^(7)^ showed that, in comparison with the IMRT, the three‐dimensional conformal proton therapy reduced the dose to the bladder, rectum, small bowel, and pelvis, while providing adequate target coverage for high‐risk prostate cancer. Zhang et al.^(10)^ did the retrospective study on ten prostate cases, comparing the IMRT and double‐scattered proton therapy. Their study reported the proton advantage for both the rectum and bladder in the low‐dose region (<50% of the target dose), whereas IMRT was better at sparing rectum and bladder >50% of the target dose. Vargas et al.^(13)^ showed that proton therapy reduced the dose to the dose‐limiting normal structures while maintaining excellent target volume coverage when compared to the IMRT.

From above mentioned studies^7^, ^10^, ^13^ and the results present in our study, it can be seen that proton beams could have potential dosimetric advantages over photons; however, further studies are warranted to determine the dosimetric quality of proton beams, especially comparing USPT with VMAT for large number of cases, which may involve high‐risk prostate cancer. Recently, Fogliata et al.^(33)^ performed a dosimetric comparison between VMAT with different dose calculation algorithms and protons for soft‐tissue sarcoma radiotherapy. Their study showed that proton plans achieved better PTV dose homogeneity, and smaller OAR volume received medium‐/low‐dose levels when compared to the VMAT plans. Fogliata and colleagues also reported the discrepancy (∼5%) in the VMAT dose distributions calculated with the dose‐to‐water option when compared to the ones computed as dose‐to‐medium option. For the VMAT planning in our study, we have used AAA, which has dose‐to‐water option to compute the dose. Recently, it has been reported that Acuros XB, a new photon dose calculation algorithm available in the Eclipse TPS, is more accurate than the AAA when heterogeneous media are involved along the photon beam path.^34^, ^35^, ^36^ Since Acuros XB is not currently available in the Eclipse TPS that was used for this project, we were unable to compute the treatment plans using Acuros XB. In the future, we plan to compare the results of proton plans with that of VMAT plans computed by Acuros XB, which has dose‐to‐medium calculation option to compute the dose.

## V. CONCLUSIONS

Dosimetric quality of two relatively new treatment modalities (VMAT and USPT) was investigated. The results presented in this study showed that the combination of lateral and oblique fields in USPT planning could potentially provide a dosimetric advantage over the VMAT for the prostate cancer involving a metallic hip prosthesis.
